# General practitioners’ and women’s experiences of perimenopause consultations: A qualitative evidence synthesis protocol

**DOI:** 10.12688/hrbopenres.13908.1

**Published:** 2024-06-24

**Authors:** Laura-Jane McCarthy, Aoife O'Mahony, Aisling Jennings, Sheena M. McHugh

**Affiliations:** 1 School of Public Health, University College Cork, Cork, T12 XF62, Ireland; 2 Department of General Practice, University College Cork, Cork, T12 XF62, Ireland

**Keywords:** Qualitative Evidence Synthesis, perimenopause, women, general practice, general practitioners, meta-ethnography

## Abstract

**Background:**

Perimenopause precedes menopause and can cause a myriad of symptoms for women. General practitioners (GPs) are frequently the first contact for perimenopausal women with symptoms. However, women express feeling dissatisfied with the consultations they have with their GPs for perimenopausal symptoms. Moreover, diagnostic difficulties can make these consultations challenging for GPs. Despite these challenges, research to date has focused on menopause, not the transition to menopause. To date, no evidence synthesis has examined how women experience perimenopause consultations, nor how GPs experience providing care to these women.

**Objective:**

To systematically search, collate, and appraise the qualitative literature to understand general practitioners' and women's experiences of perimenopause consultations and examine how treatment decisions are made during consultations.

**Methods:**

A meta-ethnography will be conducted following Sattar *et al.* (2021) guidelines for conducting a meta-ethnography as developed by Noblit and Hare (1988). Seven databases will be systematically searched. To be included, studies must report on the experiences of either general practitioners and/or perimenopausal women and published since 2014 to capture the most up-to-date evidence. Quality assessment will be conducted using CASP (Critical Appraisal Skills Programme) tools. A GRADE-CERqual (Confidence in the Evidence from Reviews of Qualitative research) will be conducted to assess the confidence of the findings. PROSPERO registration number CRD42024520537.

**Discussion:**

Findings will provide new and useful insight into how GPs and women experience consultations for perimenopause and how decisions are made during these consultations.

## Introduction

Menopause refers to the absence of a menstrual period for 12 consecutive months ^1^ as a result of the loss of ovarian follicular activity ^2^. The transition to menopause is often gradual and may be first recognized by irregularities and/or changes to the menstrual cycle as a result of greater fluctuations of sex hormones compared to premenopausal menstrual cycles ^3^. When such changes are observed a woman is said to be perimenopausal. The terms “perimenopause” and “menopausal transition” are often used interchangeably ^4^. Perimenopause precedes menopause and is the time that spans from a woman’s mature reproductive state to 12 months after her last menstrual period ^5, 6^. The majority of women experience menopause between 45 and 55 years of age ^6^ with the median onset of perimenopause at 47 years ^7^. The median length of time a woman may be perimenopausal is four years or more ^7^.

There are a myriad of menopausal symptoms reported by women during perimenopause ^8^. An estimated 20% of women who transition to menopause do so with no significant symptoms ^9^ or without the need for medical attention ^10^. However, this is not always the case, and many women experience symptoms that affect their quality of life. The 2023 state-of-the-art review by Duralde *et al.* ^11^ estimates that 60–86% of perimenopausal and menopausal women seek medical attention for their symptoms. Some of the most reported symptoms include but are not limited to altered mood, and sleep disturbances ^12^. Approximately 80% of perimenopausal women experience vasomotor symptoms which often increase in frequency in the later perimenopausal stage ^13^. Vasomotor symptoms such as hot flushes and/or night sweats are considered significant indications for menopause hormone therapy (MHT) in women without contraindications ^14^.

Research conducted in the UK in 2022 evaluated perimenopausal women’s attitudes and knowledge of menopause and found some women felt bewildered that they had reached midlife with a significant lack of awareness or knowledge of perimenopause likening it to a “hidden phenomenon” ^8^ ^(p16)^. Furthermore some women, on seeking advice for symptoms have expressed that their healthcare provider has dismissed or invalidated their concerns ^8, 15^.

Perimenopause consultations can be complex and challenging interactions for general practitioners ^16^ for numerous reasons. As mentioned, perimenopause can be associated with varied physical and psychosocial symptoms, that can be difficult to assess and address during short healthcare appointments. This may be especially true during appointments where there is a need to prioritize other health issues or rule out serious pathologies ^16^ as not all symptomology experienced by women at this stage of life are attributable to perimenopause ^17^. Diagnosing menopause is generally considered a straightforward diagnosis, in women aged 45 years and over who have not had a menstrual period for more than 12 months ^14^. However, diagnosing perimenopause is less straightforward, as some women may continue to have normal menstrual cycles, even up to the final menstrual period ^18^. Generally perimenopause is clinically diagnosed based on irregularities in the menstrual cycle and/or the presence of menopausal symptoms ^4, 19^. The National Institute for Health and Care Excellence (NICE) has published guidelines ^4^ on menopause management and diagnosis to guide healthcare providers by providing evidence-based information on the diagnosis and management of menopause and perimenopause.

Perimenopause and menopause have garnered much media attention in recent years, peaking in 2021–2022 ^20, 21^ with work-related and healthcare-related policy responses in various countries, including the United Kingdom and Ireland ^22, 23^. It has been said that increased media attention has empowered women to discuss their experiences more openly and seek medical help, increasing requests for prescriptions for hormone replacement therapy from GPs ^24^. There has been concern expressed by some GPs in the UK, that recent media attention has not only resulted in a significant increase in women seeking HRT but also younger women requesting blood tests ^25^ that may not be clinically useful ^18^.

To date, evidence syntheses generally collate evidence that reports perimenopause as part of menopause and not as a standalone unique experience ^26, 27^. For example, Ayers *et al.*’s systematic review ^28^ examined the relationship between attitudes towards menopause and symptom experience and found that the perimenopausal stage is the most difficult stage for women, attributable in part to the bodily changes experienced during this stage. To date, no evidence synthesis has examined how women experience perimenopause consultations, nor how GPs experience delivering this care. Synthesizing experiences from multiple perspectives will provide a more holistic understanding of perimenopause consultations which is important to ascertain what works well in practice and areas that may need improvement.

Therefore, the objective of this qualitative evidence synthesis is to synthesize primary research on how general practitioners and women experience perimenopause consultations and to construct a new line of argument based on the literature. This qualitative evidence synthesis intends to understand how general practitioners experience treating and managing women during consultations for perimenopausal symptoms. It also intends to understand how women experience receiving this care.

The review questions are:

i. What are general practitioners' and women’s experiences and perceptions of perimenopause consultations?
ii. How are decisions regarding treatment made during perimenopause consultations from the perspective of both general practitioners and women?

## Methods

This protocol describes a systematic review of qualitative research on GP’s and women’s experiences of perimenopause consultations in general practice. The systematic review has been prospectively registered with the International Prospective Register of Systematic Reviews (PROSPERO): CRD42024520537. This systematic review protocol adheres to the Preferred Reporting Items for Systematic review and Meta-Analysis Protocols (PRISMA-P) reporting guidelines ^29, 30^.

For this systematic review the meta-ethnographic approach of Noblit and Hare ^31^ as described by Sattar ^32^ and colleagues will be employed. Meta-ethnography is well-established and is a commonly adopted approach in conducting qualitative evidence syntheses as it allows for holistic interpretations, by comparing and understanding the interconnection between studies ^31, 33^. Meta-ethnography is a highly interpretive inductive approach, that advances understanding of a topic by synthesizing existing qualitative studies to gain a deeper understanding or inform broader concepts ^31^. Meta-ethnography consists of seven stages or phases and includes: (1) Getting started, (2) Deciding what is relevant, (3) Reading the studies, (4) Determining how the studies are related, (5) Translating the studies into one another, (6) Synthesizing the translations and (7) Expressing the synthesis. Each step is distinct from the other but often overlaps. Sattar and colleagues’ ^32^ approach to meta-ethnography will guide this meta-ethnography. It has been selected as it provides greater clarity on stages 4–6 of the meta-ethnographic process, which are often reported as the more difficult stages of conducting a meta-ethnography due to lack of guidance ^32^. This meta-ethnography will be reported in accordance to the eMERGE guidelines ^33^. The eMERGE ^33^ reporting guidelines provide structure and guidance for researchers conducting and reporting a meta-ethnography ^32^. High-quality meta-ethnographies have the potential to inform clinical guidelines and policy ^33^, as such this review will be guided and reported according to the eMERGE reporting guidelines to ensure transparency, trustworthiness, and completeness of this review.

### Phase 1: Getting started

This stage of meta-ethnography involves identifying an area of interest and providing a rationale for conducting the study. To the best of our knowledge, through preliminary searching, no meta-ethnography has synthesized general practitioners' and women’s lived experiences of perimenopausal consultations in general practice. A meta-ethnography was chosen as the most suitable approach to enable the development of a conceptual understanding of the experiences of perimenopausal consultations as experienced by both GPs and women in the context of general practice. Given the surge of attention given to menopause and perimenopause in the media ^20, 21, 34^ as well as primary qualitative research ^25, 35– 37^ conducting this qualitative evidence synthesis is timely. As recommended ^32, 38^ a research team with suitable expertise and skills has been established to conduct this meta-ethnography.

### Phase 2: Deciding what is relevant

Once the topic of interest has been established, the next four steps as described by Sattar *et al.* ^32^ will be completed:

***Phase 2a. Defining the focus of the synthesis***

Finding the balance between having a qualitative evidence synthesis with a broad scope, yet a focus that provides an appropriate but manageable number of studies can be a challenge in the meta-ethnographic approach ^32^. It is suggested that if there is little literature published on the chosen topic, a wider search of the literature should be conducted ^39^. Given the paucity of research on the phenomenon of perimenopause in isolation from menopause, it has been decided that the search strategy should be wider than perimenopause alone, so as not to miss research that reports perimenopause as a theme of menopause. Moreover, with such varied definitions of perimenopause and pre-menopause in the literature as highlighted by Ambikairajah *et al.* (2022) ^40^ it has been decided that the best approach to disentangle studies that report perimenopause as a concept of menopause is to search for perimenopause more broadly.

***Phase 2b: Locating relevant studies***

Currently, there is little consensus on how studies should be included in meta-ethnographic synthesis, nor is there agreement regarding the necessity of exhaustive literature searching ^39^. As there is little qualitative research that explores perimenopause consultations from the perspective of GPs and women our search will be expansive yet systematic. We will conduct an extensive search strategy of seven databases: Medline (via PubMed), Web of Science Core Collection, Scopus, CINAHL, Embase (Elsevier), PsycINFO and Academic Search Complete.

A comprehensive search strategy was developed guided by the Sample, Phenomenon of interest, Design, Evaluation, Research type (SPIDER) tool ^41^. A search strategy was first formulated in Medline (via PubMed) and consists of three major concepts: general practice, perimenopause and qualitative research. Search terms include a combination of Medical Subject Headings (MeSH), specific database headings, keywords, and synonymous with the following: “perimenopause” “general practice” and “qualitative research”. Boolean operators (OR, AND) were used to widen and narrow searches where appropriate. Proximity searches of keywords were also included. An academic support librarian from University College Cork was consulted and offered guidance on search strategy development.

Searches in subsequent databases will be amended as appropriate depending on database specifications. The reference list of identified studies will also be searched. The search strategy as conducted in Medline (via PubMed) is presented in Table 1 below. Given the extensive search of seven databases, grey literature sources will not be searched. Grey literature searching can be a time-consuming process with “minimally productive follow-up of the unpublished literature” ^42 (p.83)^.

**Table 1.** Search strategy conducted in Medline (via PubMed).

**#1**	"General Practice"[MeSH Terms] OR "General Practitioners"[MeSH Terms] OR "decision making, shared"[MeSH Terms]
**#2**	"General Practice"[Title/Abstract] OR "primary health care"[Title/Abstract] OR "general practitioner*"[Title/Abstract] OR "general physician"[Title/Abstract] OR "family physician*"[Title/Abstract] OR "family medicine"[Title/Abstract] OR "family practice"[Title/ Abstract] OR "primary care physician*"[Title/Abstract] OR "shared decision-making" OR "shared decision making" OR "shared decision"[All Fields] OR "shared decisions"[All Fields] OR "shared medical decision"[All Fields] OR "shared medical decisions"[All Fields] OR "shared treatment*"[All Fields] OR "shared clinical decision"[All Fields] OR "shared clinical decisions"[All Fields] OR "informed decision"[All Fields] OR "informed choice"[All Fields] OR "decision making"[All Fields] OR "decision support"[All Fields] OR "decision behavior"[All Fields] OR "choice behavior"[All Fields] OR "choice behaviour"[All Fields] OR "decision behaviour"[All Fields] OR "patient participation"[All Fields] OR "patient involvement"[All Fields] OR "participatory decision*"[All Fields] OR "collaborative decision*"[All Fields]
**#3**	#1 OR #2
**#4**	"Perimenopause"[MeSH Terms] OR "Menopause"[MeSH Terms] OR "Premenopause"[MeSH Terms] OR "Estrogen Replacement Therapy"[MeSH Terms] OR "Hormone Replacement Therapy"[MeSH Terms]
**#5**	"peri-menopause"[All Fields] OR "peri-menopause"[All Fields] OR "perimenopause"[All Fields] OR "perimenopausal symptoms"[All Fields] OR "perimenopausal complaint*"[All Fields] OR "perimenopause*"[All Fields] OR "menopause"[All Fields] OR "climacteric"[All Fields] OR "menopause transition"[All Fields] OR "menopausal transition"[All Fields] OR "HRT"[All Fields] OR "hormonal replacement"[All Fields] OR "menopause hormone therapy"[All Fields] OR "menopause transition"[All Fields] OR "menopausal transition"[All Fields]
**#6**	"hormone replacement therapy decision"[Title/Abstract:~20] OR "menopause hormone replacement therapy decision"[Title/ Abstract:~20] OR "symptoms perimenopause"[Title/Abstract:~20] OR "perimenopausal symptoms"[Title/Abstract:~20] OR "perimenopausal women"[Title/Abstract:~20] OR “perimenopause women” [Title/Abstract:~20]
**#7**	#4 OR #5 OR #6
**#8**	#3 AND #7
**#9**	"Qualitative Research"[MeSH Terms]
**#10**	"qualitative research"[All Fields] OR "mixed-methods"[All Fields] OR "mixed-methods"[All Fields] OR "questionnaire*"[All Fields] OR "focus group*"[All Fields] OR "focus group interview*"[All Fields] OR "focus group discussion*"[All Fields] OR "discourse analysis"[All Fields] OR "content analysis"[All Fields] OR "purposive sampling"[All Fields] OR "phenomenolog*"[All Fields] OR "ethnograph*"[All Fields] OR "grounded theory"[All Fields] OR "hermeneutics"[All Fields] OR "heuristic*"[All Fields] OR "narrative analysis"[All Fields] OR "thematic analysis"[All Fields] OR "themes"[All Fields] OR "thematic"[All Fields] OR "life-world"[All Fields] OR "life-world"[All Fields] OR "live experience"[All Fields] OR "case stud*"[All Fields] OR "case series"[All Fields] OR "case report*"
**#11**	#9 OR #10
**#12**	#8 AND #11

***Phase 2c: Decisions to include studies***

The SPIDER framework ^41^ will inform inclusion and exclusion criteria for screening and selection of studies. Table 2 outlines each aspect of the SPIDER tool and the corresponding inclusion and exclusion criteria.

**Table 2.** SPIDER table of inclusion and exclusion criteria.

	Inclusion	Exclusion
**S**ample	◦ General practitioners (or equivalents, must be physicians) ◦ Perimenopausal women with experience of seeking care in general practice (or equivalent)	◦ Healthcare providers that are not physicians ◦ Physicians in secondary care ◦ Post-menopausal women, women with early menopause, iatrogenic menopause
**P**henomenon of **I**nterest	◦ Perimenopause consultations ◦ Consultations for wider women’s health issues that report experiences of perimenopause	◦ Perimenopause consultations in settings other than general practice ◦ Consultations iatrogenic menopause or early menopause ◦ Consultations for menopause only (not perimenopause)
**D**esign	◦ Qualitative or mixed methods studies reporting the lived experience of perimenopause or of providing care to women with perimenopausal symptoms ◦ Primary studies reporting qualitative methods of data collection and analysis	◦ Studies that report quantitative methods only ◦ Mixed methods where it is not possible to extract qualitative data
**E**valuation	◦ Experiences of perimenopause consultations in general practice	◦ Studies that evaluate experiences of perimenopause quantitatively only ◦ Studies that do not report qualitative method of analysis
**R**esearch type	◦ English full text available ◦ Peer-reviewed literature	◦ Non-English language ◦ Grey literature, non-peer-reviewed literature, opinion pieces, editorials, protocols, reviews, theses

**Sample:** Studies must include either general practitioners or equivalents (including family physicians, and primary care physicians) and/or women with lived experience of attending general practice (or equivalent, family practice) for management of symptoms during perimenopause. Studies conducted in secondary care settings will be excluded.

**Phenomenon of Interest:** Studies that explore GPs’ and women’s lived experience of perimenopause consultations will be included. Studies that investigate consultations for other clinical conditions, menopause for example, will be included if data regarding perimenopausal symptom management can be isolated and extracted. Studies that report on wider women’s health issues that report perimenopause as a theme will be included. Studies that investigate clinical management of post-menopausal symptoms only will not be included in the review, as the phenomenon of interest is perimenopause. Studies that investigate the management of iatrogenic menopause will not be included.

The experiences of perimenopause consultations as experienced by women and GPs could include experiences of the treatment and management of symptoms including menopause hormone therapy or non-hormonal alternative medications, confidence in managing perimenopause, feelings of satisfaction or dissatisfaction, and how decisions regarding the management of symptoms are made.

**Design and Research Type:**

All primary qualitative and mixed methods studies that meet the eligibility criteria ( Table 2) will be included. Qualitative or mixed-methods studies must report primary qualitative data with recognised methods of qualitative analysis (thematic synthesis, grounded theory etc.) to be included. Studies that provide free text responses to questionnaires that have been analyzed qualitatively will be included. Studies that employ quantitative methods only will be excluded. Mixed methods studies without any recognised qualitative components will be excluded. The year of publication will be restricted to studies published after 2014 to include the most up-to-date literature and to capture studies published after the publication of NICE Guidelines: Diagnosis and Management of the Menopause (NG23) ^4^ in 2015 for healthcare professionals who care for menopausal and perimenopausal women. Due to time constraints and availability of translation services studies must be published in the English language. Studies must be peer-reviewed to be included.

Following the completion of searching all seven databases, identified articles will be imported into the bibliographic software EndNote - Clarivate ^TM^ and duplicates removed. Articles will then be exported to Rayyan – Intelligent Systematic Review - Rayyan a systematic review software tool. Further deduplication will be carried out before the screening of articles. Title/abstract screening and full-text screening will be conducted by two reviewers (LJM and AOM) independently. Where consensus cannot be reached through discussion a third review author will be consulted (AJ). A PRISMA ^43^ flow diagram will be provided to illustrate the screening and inclusion process and will outline reasons for exclusion at the full-text screening stage.

***Phase 2d: Quality appraisal***

Quality assessment will be conducted using CASP (Critical Appraisal Skills Programme) ^44^ tools for qualitative studies. Qualitative data from mixed methods studies will also be assessed using this tool. The CASP tool for qualitative studies is used widely in qualitative evidence synthesis and consists of ten questions that consider the methodological aspects of a qualitative research study ^45^. No studies will be excluded based upon quality appraisal as studies with a lower quality appraisal rating can still make a valuable contribution to qualitative evidence synthesis ^32^. Quality appraisal will be assessed by the main review author and a random sample of 20% will be checked by a second review author. Studies with lower-quality assessment scores will be included but will be highlighted in methodological limitations. Furthermore, a quality appraisal is a required step that must be completed before applying the Grading of Recommendations Assessment, Development and Evaluation-Confidence in Evidence from Reviews of Qualitative Research (GRADE-CERQual) tool ^32^.

### Phase 3: Reading the studies

Included studies will be read in-depth, with repeated reading. First order constructs (participants’ quotations) and second order constructs (authors’ themes) presented in the results or the discussion of included primary studies will be considered the raw data for this meta-ethnography ^46^. PDFs of included studies will be uploaded at this stage to NVivo - Lumivero 14 to facilitate coding of conceptual findings as they appear in the studies. Contextual data will be extracted in a data extraction form in a Microsoft Excel spreadsheet and will include author, year of publication, country of origin, study aims and objectives, study design, study setting, sample, characteristics of participants, data collection methodology, coding approaches, analysis, themes, and subthemes.

### Phase 4: Determining how the studies are related

During this stage studies will be “put together” ^31(p.28)^ through constant comparison a commonly used method in qualitative research ^47^. The relationships between studies and key concepts of included studies will be explored. A list of themes will be created to allow for comparison and identify reoccurring commonalities and concepts between studies. Concepts should not just describe the data but should explain it. A concept is a ‘meaningful idea that develops by comparing particular instances’ ^46^ ^(p. 7)^

### Phase 5: Translating the studies into one another

Concepts from each paper will be compared to identify the presence of similarities, or the absence of similarities. In doing so, it will be possible to identify differences or commonalities between concepts and will enable us to further organize them into conceptual groupings, thus creating third order constructs. Codes that have been created will be sorted into categories based on common concepts and labeled using appropriate terms that capture the key concepts that contain and express relationships that exist between and within categories. Reference to study characteristics (completed during stage 3: reading the studies) is important at this stage to give context to comparisons and to the papers themselves ^32^.

### Phase 6: Synthesising the translations: as described by Sattar *et al.* ^32^ this phase will be divided into the following stages

***(a) Reciprocal and refutational synthesis***

Third order constructs, i.e. the review authors’ interpretation of first and second order concepts will be established. At this stage studies are no longer viewed as individual studies but as a whole, in an attempt to develop a conceptual framework that may explain the phenomenon of interest ^48^. This stage involves ascertaining if studies are similar to allow for reciprocal translation synthesis or refutational synthesis whereby studies refute each other.

***(b) A line of argument synthesis***

The themes that are common across the studies will be summarized and juxtaposed to interpret a line of argument synthesis. This process will enable ‘making a whole into something more than the parts alone imply’ ^31^ ^(p.28)^ As this QES aims to synthesize the experiences of general practitioners and women, two separate syntheses will be conducted. This phase will be conducted by the main study author (LJM) and findings will be merged to create the final line of argument. Findings will be presented to the wider research team and reviewed.

### Phase 7: Expressing the synthesis

To ensure transparency and robustness in conducting this meta-ethnography the eMERGe ^33^ reporting guidelines will be adhered to, and a record will be provided. Findings will be disseminated through publication in a peer-reviewed journal and will also be presented at academic conferences.

### Confidence

A GRADE-CERQual assessment will be conducted to assess the confidence of our review findings based on methodological limitations, data adequacy, coherence and relevance ^49^. The iSoQ :: Grade-CERQual, a free online tool will be used to assist conducting and applying the GRADE-CERQual approach to findings of this qualitative evidence synthesis. The iSoQ tool ^50^ will be used to present a summary of qualitative findings table. Applying the GRADE-CERQual approach to findings that emerge from a meta-ethnography is considered a challenging process and is not well described in the literature ^51^, nonetheless, it is also considered an important step.

### Reflexivity statement

It is important to acknowledge the review authors' expertise and background, to be transparent in how these may influence findings, thus increasing rigor ^52^. LJM is a full-time PhD candidate, with a background in general nursing with a strong interest in women’s health and health equity. As the main review author, LJM will keep a journal throughout all stages of the review process, to keep note of decisions made and why decisions were made. Discussions with the wider review team will also be documented throughout. AOM is a postdoctoral researcher with a background in psychology and a strong interest in evidence synthesis and research integrity. She has not previously worked on research regarding women’s health. AJ is an academic GP. Within her clinical practice, AJ has an interest in female health. SMH is a health services researcher with a background in health psychology and interest in implementation science. SMH has carried out research on the management of chronic conditions in general practice however she has not previously conducted research on women’s health topics.

## Discussion

### Strengths and limitations

To the best of our knowledge, this review will be the first qualitative evidence synthesis to present the experiences of both women who have attended general practice for the treatment of perimenopausal symptoms and the GPs managing them. Moreover, there is no QES that gathers the evidence on experiences of GP delivering perimenopausal care. Given the dearth of research published specific to perimenopause in the context of general practice, results from this QES will add to the empirical literature and generate evidence for GPs, women, and researchers. The findings of this QES will provide a greater understanding of how GPs and women experience perimenopause consultations, aspects of these consultations that work well, and areas that may need improvement. It is important to acknowledge that meta-ethnography does have limitations. It should be acknowledged that findings from this meta-ethnography will offer just one interpretation and given the subjective nature of this methodology, the representativeness of findings may also be affected ^32^. Nonetheless, despite its challenges, meta-ethnography is invaluable in providing evidence to support practice and policy ^33^.

## Data Availability

No data are associated with this article Open science framework: General practitioners’ and women’s experiences of perimenopause consultations: A qualitative evidence synthesis protocol, https://doi.org/10.17605/OSF.IO/AWCF2 ^30^
