# Supplementary material for: General practitioners’ and women’s experiences of perimenopause consultations: A qualitative evidence synthesis protocol
Source: HRB Open Res. 2024 Jun 24;7:39. [Version 1] doi: 10.12688/hrbopenres.13908.1 (PMC11605171; doi:10.12688/hrbopenres.13908.1)
Supplement: Peer review history

# Reviewer report: Reviewer response for version 1

**DOI:** 10.21956/hrbopenres.15254.r40987

**Published:** 2024-11-28

**Authors:** Muhammad Mohsin Rana, Sana Javed

**Affiliations:** 1 Medicine, Niazi Medical and Dental College Sargodha, Sargodha, Punjab, Pakistan

The term perimenopause covers the symptoms before, during and after the menopause, starting from the time when cycles start getting abnormal through the one year after complete stopping to the period long after cessation, the hot flushes, sweating, fluctuating blood pressure and mood swings may come much later. management options include non hormonal therapies and SSRIs antidepressants.

 The methodology section is very good.

 I would love to see the complete paper.

 This article has identified a very important but still neglected issue. Identifying Perimenopasal symptoms becomes very important for those females who had hysterectomy for any other reason and had almost forgotten the menstrual cycle. for them it becomes less likely to relate these symptoms with menopause. As general practitioners are the first contact professional persons, you have very rightly focused on them for the identification of the problem and at the same time suggesting solutions. I would suggest adding a few comments about associated wide swings in Blood Pressure associated with vasomotor symptoms which may lead to unnecessary work up and prescription of antihypertensive drugs. Associated anxiety, depression and mood swings can seriously impair quality of life in these females, this needs to be part of every assessment of menopause, few comments may be added on it.

Is the study design appropriate for the research question?

Yes

Is the rationale for, and objectives of, the study clearly described?

Yes

Are sufficient details of the methods provided to allow replication by others?

Yes

Are the datasets clearly presented in a useable and accessible format?

Yes

Reviewer Expertise:

Public Health and Patient Education, Toxicology and Emergency Medicine, Respiratory and Pulmonary Care

We confirm that we have read this submission and believe that we have an appropriate level of expertise to confirm that it is of an acceptable scientific standard.

# Reviewer report: Reviewer response for version 1

**DOI:** 10.21956/hrbopenres.15254.r41188

**Published:** 2024-09-20

**Authors:** Nikki Hayfield

**Affiliations:** 1 University of the West of England, Bristol, England, UK

Thank you for the opportunity to review this paper reporting the protocol on GP and women’s experiences of peri/menopause consultations.

 There are some areas where the authors could make some further clarifications or where I had thought that the authors might like to consider as they develop their review.

 It would be useful to capture in the abstract some nuance that you touch upon in the paper itself around how menopause and peri/menopause may be used interchangeably. In the abstract the statement you make is that: ‘research to date has focused on menopause, not the transition to menopause’ – but precisely because the terms have been used interchangeably this is slightly misleading (e.g., menopause has been taken as a catch all term for perimenopause *and *menopause, hence some of the existing menopause research will include aspects of transition to menopause). However, perimenopause may not be as easy to separate out from menopause to the extent that it can be understood as an entirely ‘unique experience’ especially given the uptake in HRT and as women near the end of peri/menopause.

 From my position as a social psychologist, aspects of the paper felt focused perhaps a little too heavily on the medical aspects of perimenopause. There are not only vasomotor ‘symptoms’ during this time but also a myriad of potential psychological impacts which are often considered as important a part of experiences during this time-  and may also be explicitly relevant when women are consulting their GPs (e.g., anxiety, depression, brain fog) both in terms of them being problematic symptoms and in how they feel about the consultation when experiencing these types of issues..

 I have most commonly seen menopause hormone therapy referred to as hormone replacement therapy (HRT) – although I have heard of critiques of the term HRT and a turn to abbreviating this to hormone therapy. I wonder why the authors used the term MHT?

 Consultations may be complex and challenging for GPs, as you note, and so too can they be for women. It is the case that many women report having little knowledge of peri/menopause, but it may also be that women are becoming more aware through a recent increase in mainstream media and the cultural interest in peri/menopause. It is also likely that many women have to educate themselves in order to be taken seriously or heard by their GP and healthcare professionals. Further, they may well be having to persist to be taken by GPs and may be turning to private consultations as a last resort to be heard and supported. Finally, not all women will necessarily be seeking HRT (although some will be) but rather be seeking validation, advice, or alternative ways of managing peri/menopause.

 It’s a particularly important point that not all symptoms during peri/menopause or attributed to peri/menopause are necessarily attributable – thank you for highlighting this.

 There is increasingly a sense that may not yet be evidenced in published data that peri/menopausal experiences may start from a younger age than in the past or than is currently acknowledged. This might be a consideration alongside the current NICE guidelines age criteria and women under that age being required to have blood tests – that as you note, may not be clinically useful. It may also be a part of the reason that women are dissatisfied with their care, particularly those under 45. In community spaces there is speculation that the results of tests are unreliable and women noting that they would prefer tor their doctors to consider other measures (e.g., the menopause checklist) even in those under 45.

 While both GPs and women are present during peri/menopause consultations, their experiences are quite different. I would have liked to see a stronger rationale for examining these together rather than as two separate reviews.

 My expertise does not lie in conducting qualitative evidence synthesis. However, the protocol seems clear and comprehensive and draws on recognised methods and tools.

 I could not see the term doctor included in the search terms and this would seem a sensible addition.

 Has a scoping exercise been completed to ensure there are sufficient studies to synthesise within these topic areas?

 I wondered whether you will include all those assigned female at birth (e.g., any studies with trans men and non-binary people). It would be helpful to be clear on this from the outset..

Is the study design appropriate for the research question?

Yes

Is the rationale for, and objectives of, the study clearly described?

Yes

Are sufficient details of the methods provided to allow replication by others?

Partly

Are the datasets clearly presented in a useable and accessible format?

Not applicable

Reviewer Expertise:

Peri/menopause; Qualitative research methods

I confirm that I have read this submission and believe that I have an appropriate level of expertise to confirm that it is of an acceptable scientific standard, however I have significant reservations, as outlined above.

# Reviewer report: Reviewer response for version 1

**DOI:** 10.21956/hrbopenres.15254.r41184

**Published:** 2024-07-22

**Authors:** Antony Willman

**Affiliations:** 1 University of Birmingham, Birmingham, England, UK

Is the rationale for, and objectives of, the study clearly described? Yes

 Is the study design appropriate for the research question? Yes

 Are sufficient details of the methods provided to allow replication by others? Yes

 Are the datasets clearly presented in a useable and accessible format? N/A

 I cannot really add anything more to my comments below which I drafted while reading and re-reading. The only thing I can pick up is the decision to include mixed method papers (albeit with a qualitative analysis). I would totally agree with this strategy, it’s just justifying it in light of the decision to use the framework by Sattar et al. It’s a small point but might be worth expanding on.

 I think this proposal is otherwise excellent and definitely needed, given the lack of evidence in this area.

 1.( McCarthy et al.,2024)(Ref-1)

 Please view the additional review comments in the file found here

 Page.3: I would agree. It’s a completely different heterogenous presentation. 

 page.3: In my experience, especially with psychological symptoms, it can be difficult to know the aetiology but I share this with the patient. A very well informed patient told me after we agreed trial of HRT over her anti-depressant which wasn’t helping, she had never had depression before so was surprised to be started on fluoxetine when she did wonder about the PMP. The GP at the time didn’t ask about this or consider HRT as a treatment. For some other women however, the PMP may trigger a flare of a pre-existing affective disorder. Management may then be slightly different.

 So much has changed since 2010. GPs are much better informed and possibly more willing to try HRT for cognitive decline or impairment although the evidence isn’t great. See: (Spector A et al.,. 2024 ) (Ref-2)

 page.4: I found quite a lot of survey data of which little was published. For example, this: (Newson et al., 2019)(Ref-3)

 There was clearly more to it than what appeared in print so it might be worthwhile approaching the authors directly who I think will be responsive to your request. I agree with the grey literature search otherwise. 

 page.5: Searching might want to be expanded to include common presentations of the perimenopause which aren’t always recognized. Psychological symptoms, such as anxiety and insomnia, and other physical symptoms such as dermatological complaints. Also searching for tools which aid in both the diagnosis of the PMP (Greene, Balance app), and of other related condition such as PMP depression and the use of the MENO-D scale. Might want to include the use of antidepressants as often these are prescribed when the PMP isn’t recognized. 

 page.5: Sattar et al. state that meta-ethnography should only include qual studies, not mixed method. I think MM studies should be included, as some of the examples I found included thematic analysis of survey free text data as well as further interviews +/- focus groups. For example: (Harper JC et al.,2018)(Ref-4)

 It might be worthwhile including the reasoning for this. 

 page.6: Important to recognize the impact of the WHI and Million Women Study on HRT prescribing. GPs may still have had residual skepticism in 2014 despite NICE guidelines. The availability of TD estrogen and micronized progesterone has probably been a factor increasing HRT use alongside better informed patients. 

 page.7: I would agree

 page.7: This is important. I think managing the PMP is a great example of shared decision making and using ICE to achieve this. The PMP is a lot more widely known about but not all symptoms are due to this. There may be a risk we are over diagnosing it, and potentially missing other conditions.

Is the study design appropriate for the research question?

Yes

Is the rationale for, and objectives of, the study clearly described?

Yes

Are sufficient details of the methods provided to allow replication by others?

Yes

Are the datasets clearly presented in a useable and accessible format?

Not applicable

Reviewer Expertise:

Primary care, women’s health, medical education.

I confirm that I have read this submission and believe that I have an appropriate level of expertise to confirm that it is of an acceptable scientific standard.

## References

[1] : General practitioners’ and women’s experiences of perimenopause consultations: A qualitative evidence synthesis protocol. *HRB Open Research* .2024;7: 10.12688/hrbopenres.13908.1 10.12688/hrbopenres.13908.1 PMC1160517139619630

[2] : The effectiveness of psychosocial interventions on non-physiological symptoms of menopause: A systematic review and meta-analysis. *J Affect Disord* .2024;352: 10.1016/j.jad.2024.02.048 460-472 10.1016/j.jad.2024.02.048 38364979

[3] : Survey demonstrates suboptimal menopause care in UK despite NICE guidelines. *Maturitas* .2019;124: 10.1016/j.maturitas.2019.04.083 10.1016/j.maturitas.2019.04.083

[4] : An online survey of perimenopausal women to determine their attitudes and knowledge of the menopause. *Womens Health (Lond)* .2022;18: 10.1177/17455057221106890 17455057221106890 10.1177/17455057221106890 35758176 PMC9244939
